# Knee pain trajectories over 18 months in non-Hispanic Black and non-Hispanic White adults with or at risk for knee osteoarthritis

**DOI:** 10.1186/s12891-021-04284-8

**Published:** 2021-05-05

**Authors:** Alisa J. Johnson, Terrie Vasilopoulos, Staja Q. Booker, Josue Cardoso, Ellen L. Terry, Keesha Powell-Roach, Roland Staud, Daniel A. Kusko, Adriana S. Addison, David T. Redden, Burel R. Goodin, Roger B. Fillingim, Kimberly T. Sibille

**Affiliations:** 1 Pain Research and Intervention Center of Excellence (PRICE), University of Florida, Gainesville, FL USA; 2 Department of Community Dentistry & Behavioral Science, College of Dentistry, University of Florida, PO Box 100242, Gainesville, FL 32610 USA; 3 Department of Anesthesiology, College of Medicine, University of Florida, Gainesville, FL USA; 4 Department of Biobehavioral Nursing Science, College of Nursing, University of Florida, Gainesville, FL USA; 5 Department of Rheumatology, College of Medicine, University of Florida, Gainesville, FL USA; 6 Department of Psychology, College of Arts and Sciences, University of Alabama at Birmingham, Birmingham, AL USA; 7 Department of Biostatistics, School of Public Health, University of Alabama at Birmingham, Birmingham, AL USA; 8 Department of Aging & Geriatric Research, Institute on Aging, University of Florida, Gainesville, FL USA

**Keywords:** Osteoarthritis, Knee joint, Pain trajectory, Chronic pain, Ethnicity/race, Health disparity

## Abstract

**Background:**

Pain is the hallmark symptom of knee osteoarthritis (OA), and varies widely across individuals. Previous research has demonstrated both fluctuating and stable pain trajectories in knee OA using various time periods. Changes in pain assessed quarterly (i.e. 3-month intervals) in knee OA are relatively unknown. The current study aimed to investigate temporal variations in pain over a one and a half year period (18 months) based on quarterly characteristic pain assessments, and to examine differences in pain patterns by sociodemographic and baseline pain characteristics.

**Methods:**

The sample included a prospective cohort of 188 participants (mean age 58 years; 63% female; 52% non-Hispanic Black) with or at risk for knee OA from an ongoing multisite investigation of ethnic/race group differences. Knee pain intensity was self-reported at baseline and quarterly over an18-month period. Baseline pain assessment also included frequency, duration, and total number of pain sites. Group-based trajectory modeling was used to identify distinct pain trajectories. Multinomial logistic regression was used to examine associations between sociodemographic characteristics, risk factors, and pain trajectory groups.

**Results:**

Pain trajectories were relatively stable among a sample of adults with knee pain. Four distinct pain trajectories emerged in the overall sample, with the largest proportion of participants (35.1%) classified in the *moderate-high* pain group. There were significant relationships between age, education, income, ethnicity/race and trajectory group; with younger, less educated, lower income, and non-Hispanic Black participants had a greater representation in the highest pain trajectory group.

**Conclusions:**

Pain remained stable across a one and a half-year period in adults with or at risk for knee osteoarthritis, based on quarterly assessments. Certain sociodemographic variables (e.g. ethnicity/race, education, income, age) may contribute to an increased risk of experiencing greater pain.

## Background

Pain is the hallmark symptom of knee osteoarthritis (OA) and the primary reason people with knee OA seek medical care [1, 2]. Knee OA is among the leading causes of disability in older adults, with approximately 33 million adults in the U.S. diagnosed [3–5]. There is substantial variation in pain among individuals with or at risk for knee OA [6]. Multiple factors, in addition to radiographic disease progression [7, 8], influence the severity of knee OA-related pain. This is evidenced by prior research which identified distinct knee OA pain phenotypes based on neurophysiological and psychological indices [9–11]. There is also growing evidence for distinct pain trajectories within individuals with knee OA, which may help to inform clinical care. To date, the majority of studies have focused on annual assessments of pain to model pain progression, leaving critical questions regarding shorter-term changes, particularly quarterly changes in pain over one and a half years.

Recent scientific advances demonstrating broad heterogeneity in knee OA symptom and disease progression have challenged the historical perspective of OA being a slowly progressive disease characterized by increasing pain and articular joint degeneration [12–19]. A growing body of research examining pain and physical function trajectories in knee OA demonstrate pain progression is heterogeneous [16, 19–24]. The majority of studies have relied on annual assessments with follow-up ranging from 5 to 10 years, and have reported both progressive and non-progressive pain trajectories. For example, in a six-year longitudinal investigation of pain progression in persons with symptomatic knee OA, researchers found five pain trajectories ranging from “mild, non-progressive” to “severe, non-improving” [19]. Another study investigating pain progression annually over 5 years identified six unique pain trajectories ranging from “constant mild pain” to “constant severe pain”, with evidence for pain regression and progression [25]. One study identified five unique and stable pain trajectories based on annual assessments over a five-year period [16]. Despite these conflicting findings, it is evident that individuals with or at risk for knee OA vary in pain progression over time. It is likely that assessments taken more frequently might lead to more precise estimates of pain changes over time.

Evidence from a recent study assessing pain daily over a one-month time period, reported patients demonstrated relative stability in pain fluctuations [26]. However, the study was limited by the large percentage of participant drop out (approximately 50%) and by the use of descriptive statistics only. In another study investigating short-term fluctuations in OA-related pain over 26 weeks, researchers using latent class growth analysis, identified three distinct pain trajectories (i.e., “high, persistent pain”, “moderate, persistent pain”, and “low pain, improving”) [27]. However, this study was also limited by a high dropout rate (20%), and relatively short data collection period. While appealing, daily assessments are not feasible to capture changes in pain over a longer time span, which is critical for understanding the nature of symptom development. For example, researchers have noted that significant symptom progression can be seen in individuals with knee OA over a 1–2 year period [28–33]. Given that OA-related pain can be experienced as persistent or intermittent, with ongoing fluctuations in intensity, longitudinal studies using annual or bi-annual assessments may not fully capture clinical pain variability [18], and daily assessments place undue burden on patients. Therefore, identification of pain trajectories in knee OA based upon quarterly assessments may prove optimal by balancing the benefit to burden ratio [34].

Furthermore, despite evidence for associations between sociodemographic factors and pain trajectories [18, 34], little is known about the relationship between ethnicity/race and pain progression in knee OA. Previous research has demonstrated considerable health disparities in knee OA, with African Americans/non-Hispanic Blacks (NHBs) reporting greater overall levels of clinical pain and physical disability [21, 35–37], and experimental pain sensitivity [10, 37, 38]. Over a nine-year period of annual assessments, researchers found that African Americans (AAs) reported significantly higher pain at baseline and each measurement time-point compared to Whites (WHs) [21]. Furthermore, AAs reported more pain variation over time than WHs, yet this variability failed to reach a statistically significant difference. Overall, there was little year-to-year change in mean reported pain, except for the first year in which AAs showed a clinically significant decline in pain from baseline to 12 months [21]. Further examination of pain fluctuations using a more granular approach are warranted to better understand how pain progression influences disparities in knee OA. It is possible that greater baseline clinical pain predisposes individuals to a more severe pain trajectory, which if identified early through more frequent assessments, would improve understanding and help inform interventions to improve health outcomes.

The objectives of this study were to: (1) identify distinct pain trajectories of individuals with or at-risk for, symptomatic knee OA based on quarterly assessments over an 18-month period; 2) examine differences in sociodemographic factors, including ethnicity/race, across identified pain trajectories; and, 3) examine baseline pain characteristics among identified pain trajectory groups in NHB and NHW individuals, with or at-risk-for knee OA. We hypothesized that there would be heterogeneity (i.e. different patterns) of pain trajectories in the sample, and that these patterns would be associated with sociodemographic variables and clinical pain characteristics.

## Methods

### Study design

The current study is a secondary analysis of data from the *Understanding Pain and Limitations in Osteoarthritic Disease-2 (UPLOAD-2),* an ongoing, prospective multisite study that aims to elucidate the biopsychosocial mechanisms underlying ethnic/race group differences in knee pain between non-Hispanic Black (NHB) and non-Hispanic White (NHW) adults with or at risk for knee osteoarthritis (OA). The data set allows for the investigation of pain trajectories across 18 months using seven quarterly (3-month) pain assessments. The study was conducted at the University of Florida (UF) and the University of Alabama (UAB). Full study methods have been previously published [39, 40]. The study was approved by Institutional Review Boards at UF and UAB and was completed following the institutionally approved study protocol. Findings are presented in accordance with STROBE reporting guidelines.

### Participants

Participants from the *UPLOAD2* parent study were included in the current analysis if they had completed at least one quarterly health assessment and other measures of interest. This resulted in a sample of 188 individuals who were: (1) between 45 and 85 years of age, (2) self-identified as non-Hispanic Black (NHB) or non-Hispanic White (NHW), (3) having or being at risk for unilateral or bilateral symptomatic knee OA based upon American College of Rheumatology criteria [41], (4) able to complete multiple sessions, and (5) able to read and speak English [42]. All participants screened positive on a brief questionnaire that has previously shown 87% sensitivity and 92% specificity for radiographically confirmed symptomatic knee OA [43]. As the parent study was designed to evaluate progression of OA-related symptoms, a cohort of individuals were enrolled with a broad range of knee pain characteristics. Individuals were defined as at-risk of developing knee OA if they responded positively to the knee OA screening questions [43] but did not demonstrate radiographic joint degeneration (i.e., Kellgen- Lawrence grade ≥ 2) [44]. The broad inclusion criteria provides the opportunity to better understand factors associated with knee pain progression rather than OA pathophysiology itself. Exclusion criteria included: (1) prosthetic knee replacement or other clinically significant surgery to the arthritic knee; (2) heart disease, congestive heart failure, or history of acute myocardial infarction; (3) peripheral neuropathy; (4) systematic rheumatic disorders (e.g., rheumatoid arthritis, systemic lupus erythematosus, and fibromyalgia; (5) chronic daily opioid use; (6) neurological diseases such as Parkinson’s, multiple sclerosis, stroke with loss of sensory or motor function, or uncontrolled seizures; (7) greater pain in body sites other than knee; (8) hospitalization within preceding year for psychiatric illness; or (9) pregnant (positive human chorionic gonadotropin urine test) or breastfeeding. Participants were recruited from the community through posted fliers, radio and print media, orthopedic clinic recruitment and word-of-mouth referral, between August 2015 and May 2017. Participants provided written informed consent at the baseline visit, prior to study procedures. Following the baseline session, participants were informed that they would receive prompts via email and/or phone, to complete quarterly health assessments. Participants were compensated up to $300 for their involvement.

### Procedures

Participant eligibility was determined through an initial telephone screening. One to two weeks following screening, enrolled participants completed an in-person health assessment session (HAS) which included informed consent, pain history questionnaires and a bilateral knee joint evaluation by the study’s rheumatologists or nurse practitioner. Participants were classified as either having, or being at risk for knee OA. After the examination, participants were asked to complete online questionnaires assessing self-reported baseline knee pain symptoms. Approximately 3 months following baseline data collection, participants completed a quarterly self-reported knee pain assessment via their preferred mode of responding (online, paper, telephone), which continued to be distributed each quarter over the 18-month period, for a total of seven assessment points.

### Measures

#### Sociodemographic and health characteristics

Participants self-reported age, sex, ethnicity/race, highest education level, household income, during the telephone screening. BMI and Kellgren-Lawrence scores were assessed at baseline.

#### Baseline pain characteristics

Clinical pain was assessed at baseline (prior to quarterly pain assessments), across several domains important to understanding chronic pain trajectories [45, 46]. These included: (1) knee pain frequency (“On average, how many days per week do you experience pain in your knee?”); (2) knee pain intensity (worst, average, current); (3) knee pain duration (“For how long have you been experiencing pain in your knee?”); and, (4) total number of painful body sites. Knee pain frequency of more than 5 days per week was classified as “experiencing knee pain on most days”. Knee pain intensity was assessed using three items from the Graded Chronic Pain Scale (GCPS) [47], specific to knee pain (i.e., worst and average pain over the past 6 months, and current pain), rated on a 0 (“no pain”) to 10 (“pain as bad as could be”) numeric rating scale (NRS). These ratings were averaged and multiplied by 10 to generate a characteristic knee pain intensity score [47]. The NRS has been shown to be a reliable (ICC = 0.95) and valid (*r* = 0.94, visual analog scale) measure of OA pain [48, 49]. Knee pain duration was categorized as: (1) less than 6 months; (2) 6 months to 1 year; (3) 1 to 3 years; (4) 3 to 5 years; or, (5) more than 5 years. The total number of painful body sites was calculated as the sum of self-reported painful body sites reported. Participants could indicate if pain was experienced on the left, right, or both sides across 14 body sites. Items were coded as follows: hands (right or left = 1, both = 2); arms (right or left = 1, both = 2); neck (right or left = 1, both = 2); shoulders (right or left = 1, both = 2); head/face/jaw (right or left = 1, both = 2); chest (right or left = 1, both = 2); stomach (right or left = 1, both = 2); pelvis (right or left = 1, both = 2); upper back (right or left = 1, both = 2); lower back (right or left = 1, both = 2); knees (right or left = 1, both = 2); legs (right or left = 1, both = 2); feet (right or left = 1, both = 2), and/or 1 ‘other’ body region (free response; right or left = 1, both = 2, and summed to produce a total score with a range from 0 to 28. These four variables (pain frequency, pain intensity, pain duration, and pain sites) were summarized using descriptive statistics and compared across identified pain trajectory subgroups [45, 46].

The Western Ontario and McMaster Universities Osteoarthritis Index (WOMAC) [50], was administered at baseline to assess knee OA-related symptoms in the past 48 h. The three subscales of the WOMAC (i.e., pain, stiffness, and physical functioning) were summed to create a total symptom burden score ranging from 0 to 96 (i.e., WOMAC Global score), which was used for analysis. Higher scores indicate greater overall symptom burden. The WOMAC is a well-validated measure of pain and function in lower extremity OA [50, 51].

#### Quarterly pain assessment

Participants completed quarterly self-reported knee pain assessments every 3 months using the GCPS to assess pain intensity (“knee pain at its worst in the past week”; “knee pain on average in the past week”; and “knee pain right now”), each rated on a 0 (“no pain”) to 10 (“pain as bad as you can imagine”) NRS. NRS ratings were averaged to generate a knee pain intensity score ranging from 0 to 10 [47], which were used in to identify pain trajectories in the following analysis.

## Statistical analysis

Analyses were performed in JMP Pro 14 and SAS 9.3 (SAS Institute Inc., Cary, NC). Continuous measures were summarized with means and standard deviations and categorical measures were summarized with percentages. To identify subgroups of individuals that have similar progressions (i.e. trajectories) of pain, group-based trajectory modeling (GBTM), using maximum likelihood estimation, was implemented with PROC TRAJ in SAS® software (SAS Institute, Cary, NC) [52–54]. GBTM was used to examine heterogeneity in clinical pain patterns; GBTM does not assume that all individuals in the population will follow a similar functional form of development (i.e., it does not assume one trajectory shape will fit all) [55]. Group assignment using GBTM takes a data-driven approach, with each individual clustered into the trajectory group to which they had the highest posterior probability of membership, rather than assigning individuals to groups based on traits or other subjective criteria [36]. It is important to note that these trajectory groups represent latent strata, in that individuals are following approximately the same development pattern. However, individuals do not “belong” to a group, but are assigned based on probability of membership; any given individual could have a developmental pattern that does not exactly follow the group-level trajectory.

GBTM was used to determine both the number of distinct trajectory groups and the shape of each trajectory (i.e. order of polynomial). Model-fitting followed a multi-stage iterative process. First, a one-group model was run with the highest order of polynomial (quartic), then regression terms for each order (zero-order [intercept], linear, quadratic, cubic, and quartic) were evaluated for statistical significance (*p* < 0.05). Then, additional groups were added (2-,3-,4-,5- groups) until best fitting model was determined. Bayesian information criteria (BIC) was used to identify the most parsimonious, best fitting model, (i.e. the model that has the best fit using the fewest number of trajectories) [56]. To compare model fits, Bayes factor was calculated, which is approximately 2 times the difference in BIC between 2 models (2 x [BIC more complex model – BIC simpler model]) [52]. A Bayes factor > 2 suggests positive evidence to support a meaningful change in BIC for a more complex model, with a Bayes factor ≥ 10 providing very strong evidence [52]. Additionally, to ensure stability of the model, too small group sizes of a given trajectory group are not recommended [54]. Because GBTM utilizes full information maximum likelihood estimation, participants with missing values are still included in the models. Missingness, at each time point, ranged from 29 to 55%, with higher rates at later time points. This pattern of missing data was similar between NHBs and NHWs. A majority of the sample (78.3%) had at least five time points with complete data.

The differences in age, sex, ethnicity/race and baseline pain characteristics among trajectory groups were examined using Chi-square analysis for categorical variables and analysis of variance (ANOVA) for continuous variables. Pairwise post-hoc multiple comparison between groups was estimated using Tukey’s HSD test. Welch’s correction for ANOVA was used if equal variance assumptions were not met. A multivariable nominal logistic regression was also conducted, including sociodemographic and risk factors, to determine independent predictors of trajectory group; effects summarized as odds ratio (OR) with 95% confidence intervals (95%CIs). Note, categories for some variables were combined for logistic regression analyses. *P* < 0.05 was considered statistically significant. All methods were carried out in accordance with relevant guidelines and regulations [57].

## Results

### Sample characteristics

The average age of participants was 58 (*SD* ± 7.8) years, with a majority of participants being female (63%). The sample consisted of approximately even representation of NHBs and NHWs. Nearly half of participants reported having knee pain most days and reported experiencing pain for > 5 years. NHB and NHW participants significantly differed on age (*p* = 0.005), highest education level (*p* = 0.03), income (*p* = 0.004), GCPS score (*p* < 0.001), and total WOMAC score (*p* < 0.001). See Table 1 for full sample characteristics.

**Table 1:** Descriptive Measures (*n* = 188), for Full Sample and Stratified by Ethnicity/race

Measure	Summary	Non-Hispanic Black	Non-Hispanic White
**Age**, mean year ± SD	58.0 ± 7.8	56.4 ± 6.5	59.6 ± 8.6
**Gender**, %(n)			
Female	63.3% (119)	59.8% (58)	67.0% (61)
Male	36.7% (69)	40.2% (39)	33.0% (30)
**Ethnicity/Race**, %(n)			
Non-Hispanic Black	51.6% (97)	–	–
Non-Hispanic White	48.4% (91)	–	–
**Highest Education Level,** %(n)			
Some high school	6.9% (13)	9.3% (9)	4.4% (4)
High school degree	41.5% (78)	48.5% (47)	34.1% (31)
Two-year college degree	17.6% (33)	19.6% (19)	15.4% (14)
Four-year college degree	19.7% (37)	13.4% (13)	26.4% (24)
Master’s degree	10.6% (20)	7.2% (7)	14.3% (13)
Doctoral degree	3.7% (7)	2.1% (2)	5.5% (5)
**Household Income,** %(n)			
$0 - $9999	29.3% (54)	38.3% (36)	20.0% (18)
$10,000 - $19,999	13.6% (25)	16.0% (15)	11.1% (10)
$20,000 - $29,999	14.1% (26)	18.1% (17)	10.0% (9)
$30,000 - $39,999	4.4% (8)	5.3% (5)	3.3% (3)
$40,000 - $49,999	7.1% (13)	3.2% (3)	11.1% (10)
$50,000 - $59,999	8.7% (16)	5.3% (5)	12.2% (11)
$60,000 - $79,999	8.2% (15)	6.4% (6)	10.0% (9)
$80,000 - $99,999	5.4% (10)	4.3% (4)	6.7% (6)
$100,000 - $149,999	6.5% (12)	2.1% (2)	11.1% (10)
$150,000+	2.7% (5)	1.1% (1)	4.4% (4)
**BMI**, mean ± SD	32.0 ± 7.7	32.8 ± 7.8	31.2 ± 7.5
**Study Site**, %(n)			
University of Florida	64.4% (121)	59.8% (58)	69.2% (63)
University of Alabama, Birmingham	35.6% (67)	40.2% (39)	30.8% (28)
**Kellgren- Lawrence (KL) Score**, %(n)			
0	29.3% (54)	29.5% (28)	29.2% (26)
1	15.8% (29)	8.4% (8)	23.6% (21)
2	20.7% (38)	25.3% (24)	15.7% (14)
3	16.3% (30)	16.8% (16)	15.7% (14)
4	17.9% (33)	20.0% (19)	15.7% (14)
**Baseline Pain Characteristics**			
*Frequency*: Currently experiencing knee pain on most days, % yes (n)	47.9% (90)	50.5% (49)	45.1% (41)
*Intensity*: Graded Chronic Pain Scale (0–100), mean ± SD	55.8 ± 23.1	66.9 ± 20.3	43.9 ± 19.8
*Time/Duration*:			
< 6 months	5.4% (10)	4.2% (4)	6.7% (6)
6 months to 1 year	8.0% (15)	10.3% (10)	5.6% (5)
1 to 3 years	25.1% (47)	28.9% (28)	21.1% (19)
3 to 5 years	14.4% (27)	16.5% (16)	12.2% (11)
> 5 years	47.1% (88)	40.2% (39)	54.4% (49)
*Total Pain Sites*: Number of painful body sites with pain more than not over last 3 months, mean ± SD	5.7 ± 3.6	5.8 ± 3.7	5.5 ± 3.6
**WOMAC total score**, mean ± SD	36.2 ± 19.9	42.9 ± 18.5	29.1 ± 18.9

### Group-based trajectory modeling

**Fig. 1:**
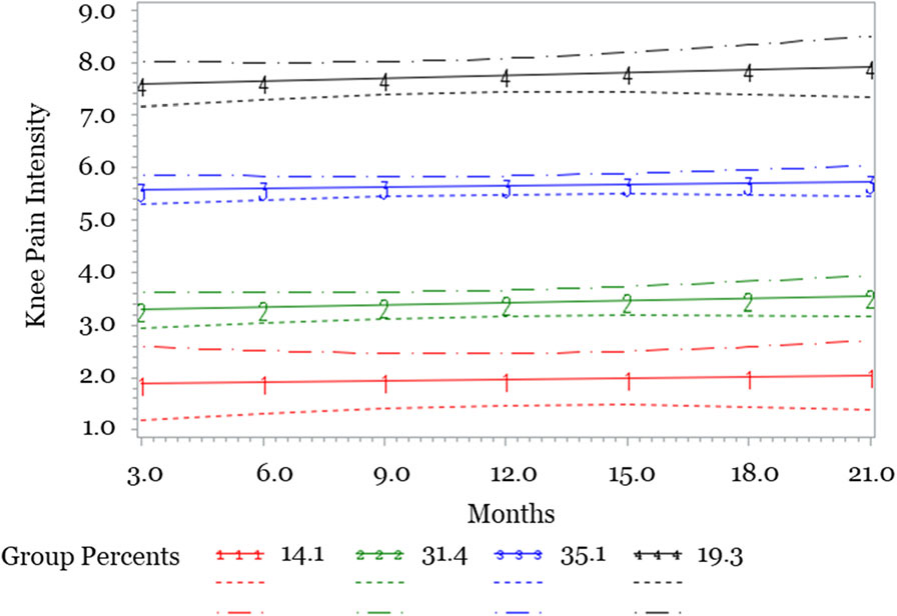
Pain trajectory groups in the overall sample over 7 quarterly (3-month intervals) assessments over an 18-month period with 95% confidence intervals. Note. Percentages indicate the proportion of the sample included in each trajectory group

Table 2 reports model fitting results for the GBTM analysis. In the overall sample, the best fitting model included 4 trajectory groups, all linear order. Trajectory groups are labeled according to characteristic knee pain intensity patterns (low, moderate-low, moderate-high, and high) (Fig. 1). The largest proportion of participants (35.1%) were in the *moderate-high* pain group, followed by 31.4% of participants in the *moderate-low* pain group. The *low* pain group had the smallest proportion of participants (14.1%), with the remaining 19.3% of participants in the *high* pain group. An overall pattern of worsening or improvement in reported pain across the 2-year study period was not observed in any of the four trajectory groups, with linear coefficients for each group the best-fitting model nor statistically significantly different from zero (0) (Table 2). Average posterior probabilities for trajectory groups were 80% or higher for all groups, with highest posterior probabilities high pain group: *low* (88%), *moderate*-*low* (87%), *moderate*-*high* (80%), and *high* (95%).

**Table 2:** Model Fitting for Group Based Trajectory Analysis

# Groups	Order	Term	Group 1 B(SE) ***p***-value	Group 2 B(SE) ***p***-value	Group 3 B(SE) ***p***-value	Group 4 B(SE) ***p***-value	Group 5 B(SE) ***p***-value	BIC	Bayes Factor
1	All quartic	Intercept	4.45 (1.24) *P* < 0.001					− 1812.5	
		Linear	0.55 (1.58) *P* = 0.77						
		Quadratic	−0.31 (0.87) *P* = 0.72						
		Cubic	0.06 (0.16) *P* = 0.69						
		Quartic	0 (0.01) *P* = 0.66						
1	All linear	Intercept	4.68 (0.2) *P* < 0.001					− 1802.6	
		Linear	0.02 (0.04) *P* = 0.49						
2	All linear	Intercept	2.9 (0.17) *P* < 0.001	6.26 (0.16) *P* < 0.001				− 1574.8	227.8
		Linear	0.02 (0.04) *P* = 0.49	0.04 (0.04) *P* = 0.33					
3	All linear	Intercept	2.76 (0.16) *P* < 0.001	5.48 (0.2) *P* < 0.001	7.5 (0.24) *P* < 0.001			−1517.3	57.5
		Linear	0.03 (0.04) *P* = 0.40	0.02 (0.04) *P* = 0.55	0.05 (0.06) *P* = 0.38				
**4**	**All linear**	**Intercept**	**1.83 (0.32)** ***P*** **< 0.001**	**3.24 (0.20)** ***P*** **< 0.001**	**5.55 (0.17)** ***P*** **< 0.001**	**7.54 (0.22)** ***P*** **< 0.001**		**− 1502.5**	**14.8**
		**Linear**	0.03 (0.06) *P* = 0.67	0.04 (0.05) *P* = 0.34	0.03 (0.04) *P* = 0.50	0.05 (0.05) *P* = 0.32			
5	All linear	Intercept	1.8 (0.32) *P* < 0.001	3.21 (0.2) *P* < 0.001	5.18 (0.21) *P* < 0.001	6.92 (0.44) *P* < 0.001	7.61 (0.27) *P* < 0.001	− 1505.5	−3.0
		Linear	0.3 (0.06) *P* = 0.66	0.05 (0.04) *P* = 0.31	0.06 (0.05) *P* = 0.19	−0.08 (0.08) *P* = 0.33	0.10 (0.06) *P* = 0.12		

Note: B(SE)➔ regression coefficient with standard error, by number of groups in model. *P*-values are for statistical significance of each regression term in model. BIC➔ Bayesian Information Criteria. Bayes factor➔ 2 x [BIC more complex model – BIC simpler model] Best-fitting model in **bold**

### Participant sociodemographic characteristics and trajectory groups

There were differences between trajectory groups for age (F_(3,184)_ = 8.3, *p* < 0.001), ethnicity/race (*χ* ^2^ = 47.5, df = 3, p < 0.001), highest education level (*χ* ^2^ = 57.3, df = 3, p < 0.001), household income (*χ* ^2^ = 54.8, df = 3, p < 0.001), BMI (F_(3,184)_ = 8.9, p < 0.001), and Kellgren-Lawrence (KL) score (*χ* ^2^ = 27.8, df = 3, *p* = 0.006) (Table 3). Participants in the *high* pain group (mean age = 54 years) were, on average, a decade younger than participants in the *low* pain group (mean age = 64 years). Non-Hispanic Black (NHB) participants were not represented in the *low* pain group and comprised three-quarters of the *high* pain group. Those with highest level of education of high school or less comprised the majority of the *high* pain (71.0%) and *moderate-high* pain (64.2%) groups. Similarly, those with lowest household income (<$20,000) comprise the vast majority of the *high* pain group (80%). BMI was significantly higher in *high* pain group compared to all other groups. Finally, the majority of the *high* pain group include participants with KL scores of 3 or higher, though, nearly 30% of the *high* pain group also included those with KL scores of 0 or 1. Interestingly, while nearly 60% of the *low* pain group included those with KL scores of 0 or 1, nearly one-third of the *low* pain group was comprised of individuals with KL scores of 3 or higher.

**Table 3:** Sample Characteristics by Pain Trajectory Group

	Low (***n*** = 22)	Moderate-Low (***n*** = 57)	Moderate- High (***n*** = 78)	High (***n*** = 31)	p	Pairwise comparisons
**Age**, mean year ± SD	63.7 ± 7.0	59.3 ± 8.8	56.8 ± 6.5	54.3 ± 6.7	< 0.001	Low > High & Moderate-High (*p* < 0.001) Moderate-Low > High (*p* = 0.014)
**Gender**, %					0.054	
Female	72.7%	66.7%	52.6%	77.4%		
Male	27.3%	33.3%	47.45	22.6%		
**Ethnicity/Race**, %					< 0.001	
Non-Hispanic Black	0%	40.4%	65.4%	74.2%		
Non-Hispanic White	100%	59.6%	34.6%	25.8%		
**Highest Education Level,** %(n)					< 0.001	
Some high school	0%	1.8%	7.7%	19.4%		
High school degree	13.6%	26.3%	56.4%	51.6%		
Two-year college degree	27.3%	12.3%	21.8%	9.7%		
Four-year college degree	31.8%	31.6%	9.0%	16.1%		
Master’s or Doctoral degree	27.3%	28.1%	5.1%	3.2%		
**Household Income,** %(n)					< 0.001	
$0 - $19,999	19.1%	19.6%	52.0%	80.0%		
$20,000 - $39,999	9.5%	25.0%	20.8%	6.7%		
$40,000 - $59,999	19.1%	25.0%	14.3%	0%		
$60,000 - $99,999	33.3%	14.3%	10.4%	6.7%		
$100,000+	19.1%	16.1%	2.6%	6.7%		
**BMI**, mean ± SD	27.9 ± 5.8	31.4 ± 5.8	31.5 ± 6.9	37.7 ± 10.5	< 0.001	High > all other groups, p < 0.001
**Kellgren-Lawrence (KL) Score**, %(n)					0.006	
0	27.3%	37.5%	27.6%	20.0%		
1	31.8%	19.6%	11.8%	6.7%		
2	9.1%	19.6%	30.3%	6.7%		
3	13.6%	12.5%	15.8%	26.7%		
4	18.2%	10.7%	14.5%	40.0%		
**Baseline Pain Characteristics**						
*Frequency*: Currently experiencing knee pain on most days, % yes	27.3%	40.4%	47.4%	77.4%	0.002	
*Intensity*: GCPS (0–100), mean ± SD	31.5 ± 12.2	45.1 ± 18.1	61.4 ± 20.3	78.5 ± 17.1	< 0.001	All pairwise comparisons p < 0.05
*Time/Duration*:					0.523	
< 6 months	4.6%	3.6%	9.0%	0%		
6 months to 1 year	9.1%	3.6%	9.0%	12.9%		
1 to 3 years	22.7%	25.0%	20.5%	38.7%		
3 to 5 years	13.6%	17.9%	12.8%	12.9%		
> 5 years	50.0%	50.0%	48.7%	35.8%		
*Pain Sites*: Number of body sites with pain, mean ± SD	4.0 ± 2.7	5.1 ± 3.2	6.1 ± 3.4	7.0 ± 4.7	0.010	High > Low, *p* = 0.015
**WOMAC total score**	16.8 ± 10.8	27.7 ± 14.9	39.2 ± 18.0	57.2 ± 16.1	< 0.001	All pairwise comparisons p < 0.05

Note: *GCPS* Graded Chronic Pain Scale, *WOMAC* Western Ontario and McMaster Universities Osteoarthritis Index. Pairwise posthoc multiple comparison between groups (for continuous measures) was estimated using Tukey’s HSD test

In multivariable nominal logistic regression analysis (Table 4) with sociodemographic and risk factors, age (*p* = 0.031), ethnicity/race (*p* = 0.012), education (*p* = 0.003), household income (*p* < 0.001), and KL scores (*p* = 0.001) remained as independent predictors of pain trajectories; odds ratios used combined low and moderate-low pain group as reference. As age increased, participants had decreased likelihood of being in high pain group (OR = 0.90, 95%CI:0.82, 0.99). Compared to NHWs, NHBs were more likely to be in high pain (OR = 5.28, 95%CI:1.5, 18.66) and the moderate-high pain group (OR = 2.57, 95%CI:1.15, 5.71). For household income, those with incomes less than $20,000 were more likely to be in high pain group (OR = 8.50, 95%CI:1.62, 44.49) and moderate-high pain group (OR = 3.36, 95%CI:1.07, 10.51). BMI (*p* = 0.143) and gender (*p* = 0.337) were not statistically significant predictors in multivariable analysis.

**Table 4:** Results from Multivariable Nominal Logistic Regression

	Moderate-High Pain Group Vs. Low/Moderate-Low Pain Group		High Pain Group Vs. Low/Moderate-Low Pain Group	
	OR	95%CI	OR	95%CI
**Age**	0.94	0.89,1	0.90	0.82,0.99
**Gender**				
Female	0.65	0.28,1.49	1.44	0.37,5.55
Male	Ref.		Ref.	
**Ethnicity/Race**,				
Non-Hispanic Black	2.57	1.15,5.71	5.28	1.5,18.66
Non-Hispanic White	Ref.		Ref.	
**Highest Education Level**				
Some high school/ High school	4.69	1.91,11.55	2.87	0.76,10.8
Two-year college degree or beyond	Ref.		Ref.	
**Household Income,**				
$0 - $19,999	3.36	1.07,10.51	8.50	1.62,44.49
$20,000 - $59,999	1.96	0.69,5.51	0.26	0.03,2.03
$60,000+	Ref.		Ref.	
**BMI**, mean ± SD	0.99	0.93,1.05	1.06	0.98,1.16
**Kellgren-Lawrence (KL) Score**				
0	Ref.		Ref.	
1	0.83	0.25,2.74	0.95	0.13,7.04
2	4.24	1.32,13.62	0.97	0.13,7.44
3 or 4	3.60	1.14,11.4	12.09	2.59,56.46

Note: OR➔ odds ratio, 95%CI➔ 95% confidence intervals

### Baseline pain characteristics and trajectory groups

There were notable differences among pain trajectory groups in baseline pain characteristics. Specifically, knee pain frequency (*χ* ^2^ = 16.6, df = 3, *p* = 0.001), knee pain intensity (F_(3,184)_ = 37.5, *p* < 0.001), and total number of pain sites (F_(3,184)_ = 3.9, *p* = 0.011) differed significantly across pain trajectory groups. Three-quarters of participants in the *high* pain trajectory group reported experiencing knee pain on most days, compared to less than half of participants in each other pain group (Table 3). Participants in *high* pain groups also reported the greatest number of pain sites and had the highest knee pain intensity scores. Participants in the *low* pain trajectory group reported the fewest number of pain sites and had the lowest knee pain intensity scores (Table 3). There were similar trajectory group differences in total WOMAC scores (Table 3), with participants in the higher pain trajectories having higher baseline total WOMAC scores (F_(3,182)_ = 33.8, *p* < 0.001).

## Discussion

The overall intention of the current study was to investigate quarterly variations in pain over an 18-month period and to examine differences in pain patterns by sociodemographic, health, and baseline pain characteristics. Four distinct pain trajectories (i.e., *low*, *moderate-low*, *moderate-high*, and *high*) emerged within the overall sample. The majority of our participants fell within the *moderate-low* and *moderate-high* pain trajectory subgroups, based on quarterly pain assessments. The *low* pain group had the lowest representation across the sample. On average, identified trajectories remained stable within individuals over time, which is consistent with previous large studies using annual assessments [16, 23, 24, 58, 59].

Identified pain trajectory groups differed across sociodemographic and health variables, including age, ethnicity/race, education, income, BMI, and KL grade. Participants in the *low* pain trajectory group were older compared to the *high* pain group. This age finding is consistent with other pain trajectory studies conducted in the US [16], but is in contrast to studies conducted outside of the US, which show significant associations between increasing age and pain [59]. It is possible that older adults with more severe pain were excluded from the current study unintentionally due to the exclusion of individuals with comorbidities, resulting in a relatively healthy sample of older adults who were not experiencing high levels of pain. Another possible explanation is that younger participants are more likely to work and engage in activities which may result in greater self-reported pain. Importantly, Non-Hispanic Blacks (NHBs) were not represented in the *low* pain group and comprised the majority of the *high* pain group. Additionally, lower education, lower income, and BMI were also associated with higher pain trajectories.

An important consideration for this sample is that NHBs were, on average, younger and reported lower education and income compared to NHW participants. Despite this potential confounding, in multivariable analysis, age, ethnicity/race, education, and income remained as independent predictors of pain trajectories. In addition to sociodemographic factors, individuals with more significant radiographic joint disease (indicated by higher KL scores) were also more likely to be in higher pain trajectory groups. This finding is consistent with the study by Wesseling and colleagues (2015) indicating a higher KL score placed individuals at a greater risk for a moderate pain trajectory [60], but differs from the study by Collins et al. (2014) which demonstrated no differences in pain trajectories based on KL scores [16].

Pain trajectory groups also differed on baseline clinical pain characteristics such that the majority of individuals in the *high* pain trajectory group reported experiencing knee pain on most days, greater knee pain intensity at baseline, and more bodily pain sites compared to the other pain trajectory groups. This supports prior research which found comorbid musculoskeletal (i.e. hip) pain [60], and baseline pain severity [25], placed individuals with knee OA at greater risk of developing a moderate pain trajectory [60]. Consistent with prior research NHB participants reported higher levels of baseline clinical pain across trajectory groups compared to NHWs [21, 35].

The current findings regarding greater pain in NHBs with knee OA are supportive of prior research indicating that NHBs/African Americans have increased risk of experiencing greater OA-related pain over time [16, 21]. However, as noted in other research, ethnicity/race is a construct associated with environmental, social, and cultural experiences that impact a number of factors influencing health including multiple chronic pain conditions, access to health care and treatment, and management strategies [37, 61]. However, the current analyses are somewhat limited in the number of variables available to fully examine the complex array of factors contributing to environmental and sociodemographic influences on pain outcomes [62]. While the mechanisms underlying ethnic/race group differences have yet to be fully elucidated, research indicates central sensitization (e.g. hyperexcitability of the central nervous system), may place some individuals with knee OA at a greater risk for increased pain severity [10, 38, 63], and further investigation of factors contributing to observed ethnic/race differences in clinical and experimental pain are underway [10, 64–66].

To our knowledge this is among the first studies evaluating pain trajectories in OA using quarterly self-reports. Prior research of pain trajectories has relied on a few large samples, with annual assessments, that may not be fully representative of NHB and NHW adults with, or at-risk-for, knee OA. Investigating changes in knee pain quarterly across an 18-month time span provides a more nuanced appreciation for trajectory patterns of knee pain symptoms that may differ from those assessed annually, and can inform the development of more accurate prognosis pathways for precision care. One study utilizing quarterly pain assessments over 2 years in hip OA classified patients into five pain trajectories and showed symptom progression over time [67]. This contrasts with the current study’s findings and may be partly explained by the differences in symptom progression between hip and knee OA [68]. The lack of observed pain progression within identified trajectories could be due in part to adaptive behaviors, coping skills, and pharmacological pain management [69, 70]. Another explanation is that knee OA may have a sustained period of symptomatic stability followed by symptom escalation [16, 18, 21]. Overall, this has significant clinical relevance in terms of prevention, rehabilitation and symptom management, warranting further investigation.

### Implications and future directions

The heterogeneity of pain identified in the current sample, and in prior studies, highlights the inter-individual differences in symptom progression over time. Understanding pain trajectories associated with knee OA can inform clinical care and best treatment practices. For example, individuals experiencing frequent, high intensity knee pain in conjunction with multisite pain may be at a higher risk of poor health outcomes, surgical complications, and may require multimodal pain management approaches [71]. Future efforts aimed at developing algorithms useful for identifying individuals at a higher risk for a greater symptom trajectory could inform the development of targeted interventions for individuals with knee OA pain. Future research examining associations between pain processing (e.g., peripheral and central sensitization), psychological factors and pain trajectories, will further our understanding of the mechanistic contributors to pain, and will help to identify other key indicators to inform clinical decision making. This could include studies into the relationship between pain self-management and temporal changes in symptoms to inform personalized medicine approaches which allow for prognosis-based treatment planning.

### Strengths and limitations

The results of this study should be interpreted in light of its strengths and limitations. This was a cohort study with equal representation of NHB and NHW participants with, or at-risk-for, knee OA which allowed for important comparisons. We used group-based multi-trajectory modelling (GBMT), which allows for the possibility of distinct subgroups that can be identified within a population [55, 72]. Our findings add novel information regarding knee pain intensity trajectories across quarterly assessments over an 18-month period, thus reducing potential bias from symptom fluctuations captured at only one time point in a given year [73]. Furthermore, use of a validated measure, the Graded Chronic Pain Scale (GCPS) to assess pain quarterly provides a more robust characterization of symptoms targeted over the past week [47], rather than measures with a shorter symptom assessment period (e.g., WOMAC-48 h).

Several limitations must also be considered, including the proportion of missing data. Also, longitudinal evaluation of group means may obscure within-person variability, and misclassification is possible. Another limitation is our smaller sample (< 200), inadequate sample sizes can bias model solutions and can make it difficult identify smaller subgroups [74]. As quarterly assessments were completed online, we cannot be sure what influence participant environment had on pain assessments. However, participants were provided with the same instructions at each assessment point to reduce potential response bias. Additionally, the use of a single pain dimension (i.e., intensity), may not provide full information regarding symptom progression and health outcomes, despite being a well-validated instrument. Also, with the exception for exclusion if an individual was taking opioids on a daily basis, we did not control for pain medication in this study. However, previous research using the *UPLOAD2* data set found that less than half of participants reported using pain medication, which did not differ significantly by ethnicity/race. And finally, our exclusion criteria may limit generalizability of our findings. Despite these limitations, this study improves understanding regarding pain trajectories in knee OA, providing the next level of evidence regarding symptom progression in knee OA by replicating findings from similar, larger studies using annual assessments in sample with more detailed, frequent assessments.

## Conclusions

Our findings provide further evidence for stable, yet heterogeneous, pain trajectories in adults with, or at-risk-for, knee OA-related pain with greater temporal resolution. Four stable knee pain trajectories were identified in this sample, and differed significantly in respect to several sociodemographic factors including age, ethnicity/race, education, and income. NHBs who were on average, younger, less educated and reported lower income compared to NHWs were more likely to report high pain levels at the baseline and were categorized in the stable *high* pain trajectory over 18 months. Taken together, our findings further confirm the stability of pain progression in knee OA and improves our understanding of pain progression and highlights the importance of considering sociodemographic characteristics in understanding pain heterogeneity.

## Data Availability

The datasets used and/or analyzed during the current study are available from the corresponding author on reasonable request.

## Abbreviations

ANOVA: Analysis of variance
BIC: Bayesian information criterion
BMI: Body mass index
CID: Clinically important difference
GBTM: Group-based trajectory modeling
GCPS: Graded Chronic Pain Scale
NHB: Non-Hispanic Black
NHW: Non-Hispanic White
NRS: Numerical rating scale
OA: Osteoarthritis
WOMAC: Western Ontario and McMaster Universities Osteoarthritis Index
